# The mitochondrial epitranscriptome: the roles of RNA modifications in mitochondrial translation and human disease

**DOI:** 10.1007/s00018-017-2598-6

**Published:** 2017-07-27

**Authors:** Markus T. Bohnsack, Katherine E. Sloan

**Affiliations:** 1 Department of Molecular Biology, University Medical Center Göttingen, Humboldtallee 23, 37073 Göttingen, Germany; 2 Göttingen Centre for Molecular Biosciences, University of Göttingen, Justus-von-Liebig-Weg 11, 37077 Göttingen, Germany

**Keywords:** RNA modification, Mitochondria, Ribosome, tRNA, Translation, Mitochondrial disease, Protein synthesis, Epitranscriptome

## Abstract

Mitochondrial protein synthesis is essential for the production of components of the oxidative phosphorylation system. RNA modifications in the mammalian mitochondrial translation apparatus play key roles in facilitating mitochondrial gene expression as they enable decoding of the non-conventional genetic code by a minimal set of tRNAs, and efficient and accurate protein synthesis by the mitoribosome. Intriguingly, recent transcriptome-wide analyses have also revealed modifications in mitochondrial mRNAs, suggesting that the concept of dynamic regulation of gene expression by the modified RNAs (the “epitranscriptome”) extends to mitochondria. Furthermore, it has emerged that defects in RNA modification, arising from either mt-DNA mutations or mutations in nuclear-encoded mitochondrial modification enzymes, underlie multiple mitochondrial diseases. Concomitant advances in the identification of the mitochondrial RNA modification machinery and recent structural views of the mitochondrial translation apparatus now allow the molecular basis of such mitochondrial diseases to be understood on a mechanistic level.

## Introduction

Mitochondria are essential eukaryotic organelles that produce the majority of cellular energy by oxidative phosphorylation (OXPHOS) and also play important roles in other cellular processes, such as apoptosis, regulating intracellular calcium levels and ageing, and in various metabolic pathways [1–4]. They are thought to originate from the endocytosis of an α-proteobacterium, which was retained by the host cell as it evolved to confer a selective advantage due to its ability to produce energy in the form of adenosine triphosphate (ATP) [5]. Consequently, all eukaryotic cells contain distinct sets of nuclear and mitochondrial genetic information and two separate protein synthesis machineries. In mammals, the mitochondrial genome is a multi-copy, circular, double stranded DNA (mt-DNA) that encodes 13 polypeptides, which are components of the OXPHOS system, as well as two ribosomal RNAs (mt-rRNAs) and 22 transfer RNAs (mt-tRNAs) [6]. Expression of the mt-DNA is essential for proper cellular function and is closely co-ordinated with nuclear gene expression as the remaining components of the electron transport chain (complexes I–IV), the ATP synthetase (complex V), and various factors required for biogenesis of the mitochondrial translation machinery are encoded within the nuclear genome, translated on cytoplasmic ribosomes and imported into mitochondria (reviewed in [7, 8]). Expression of the human mitochondrial genome is initiated by transcription of the mt-DNA from bidirectional heavy and light strand promoters (HSP and LSP, respectively) to produce two polycistronic transcripts: one containing the sequences of the two mt-rRNAs, 14 mt-tRNAs, and 10 mt-mRNAs (eight monocistronic and two bicistronic) and the other encoding eight mt-tRNAs and one mt-mRNA (reviewed in [9]). Release of the individual RNA elements is thought to be largely achieved by excision of the mt-tRNAs that directly flank the mt-rRNA and mt-mRNA sequences [10]. There are, however, several exceptions, such as the bicistronic mRNA encoding ATP6 and ATP8 that is immediately followed by the COX3 mRNA sequence, with no intervening mt-tRNA (reviewed in [11]). The mt-tRNAs then undergo further processing and aminoacylation, and the 16S and 12S mt-rRNAs, together with either mt-tRNA^Val^ or mt-tRNA^Phe^, are assembled into 55S mitoribosomes. Overviews of the pathways of mt-tRNA maturation and mitoribosome biogenesis are provided by several recent reviews [12–15], and here, we will focus on a key aspect of the maturation of mitochondrial RNAs, the introduction of chemical modifications to specific nucleotides by nuclear-encoded enzymes that are imported into mitochondria to perform this function.

RNA modifications are present in most cellular RNAs in all three domains of life, and to date, more than 100 different types of modifications have been identified [16]. The discovery of this plethora of chemical modifications in RNAs has, by analogy to the long known “epigenetic” markers in DNA, led to the introduction of the term “epitranscriptome” to collectively describe modifications in coding and non-coding RNAs. In general, RNA modifications serve to expand the chemical and topological properties of the four basic nucleotides and thereby influence the biogenesis, dynamics, stability, and function of the RNAs/RNPs that carry them. tRNAs and rRNAs are the most extensively modified types of RNA and several unique features of mitochondrial gene expression make modification in mt-tRNAs and mt-rRNAs especially important. Mitoribosomes have a high protein-to-RNA ratio compared to all other ribosomes [17], and due to minimisation of the mt-rRNA scaffold, correct folding and high stability of this structure are particularly critical to ensure the fidelity of mitochondrial ribosome biogenesis and function. Similarly, many mt-tRNAs fold into non-canonical structures [18, 19], which require additional stabilisation by RNA modifications in the core of the mt-tRNAs to ensure that they can be recognised by aminoacyl-tRNA-synthetases and function accurately in translation. Furthermore, the use of a non-universal genetic code in mammalian mitochondria (reviewed in [20]) requires the minimal set of only 22 mt-tRNAs to decode 60 different codons and the necessary decoding flexibility of the mt-tRNAs is largely achieved through the installation of complex RNA modifications in their anticodons.

In addition to these roles in maintaining the stability and functionality of the core translation machinery, the importance of RNA modifications as dynamic regulators of RNA fate has been highlighted by the identification of demethylases that can “erase” specific modifications (reviewed in [21]) and the characterisation of several proteins, termed “readers”, which recognise particular modifications in cellular RNAs [22]. Furthermore, detection of substoichiometric modification in rRNAs [23, 24] indicates that rRNA modifications may represent an important layer of translational control of gene expression (reviewed in [25]). Such dynamic regulation of gene expression is likely to be highly important in mitochondria as their function needs to be modulated to meet cellular energy demands during adaptation to changing environmental conditions and developmental cues. The disruption of mitochondrial protein synthesis impedes assembly of the components of the mitochondrial respiratory chain and is often associated with disease (see for example [26, 27] and reviewed in [28]). Due to the importance of the OXPHOS system especially in highly energy-consuming tissues, such as brain and muscle, these disorders are often collectively referred to as encephalomyopathies, but can present with a broad range of additional symptoms, including blindness, deafness, failure to thrive, and lactic acidosis. Advancements in whole-exome sequencing have revealed that such mitochondrial diseases can arise due to mutations either in the mt-DNA or in nuclear genes encoding factors that are required for assembly of the mitochondrial translation machinery. The growing inventory of such pathogenic mutations (see for example, MITOMAP (https://www.mitomap.org/MITOMAP) [29]) reveals that many occur in mt-DNA regions that are transcribed into mt-rRNAs and mt-tRNAs, or in nuclear genes encoding factors that are required for mitochondrial protein synthesis. More specifically, many mutations have been found in mitochondrial RNA modification enzymes and at or near mt-RNA sites that carry modifications, highlighting the important roles that mt-RNA modifications play in facilitating and regulating mitochondrial gene expression, and suggesting strong links between lack of mt-RNA modifications and disease.

## mt-rRNA modifications

**Fig. 1:**
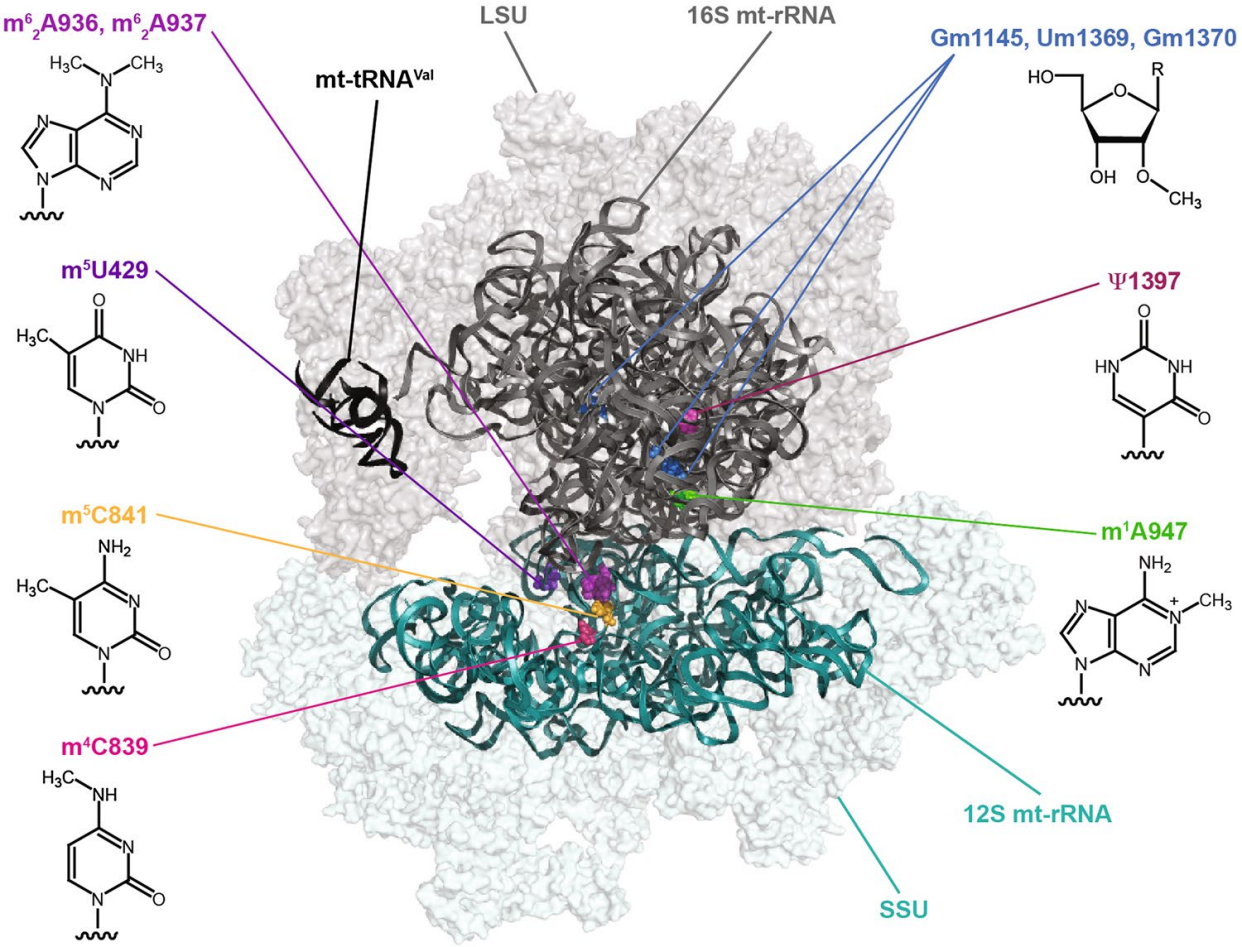
Distribution of RNA modifications on the human mitochondrial ribosome (PDB 3J9M) [30]. The small ribosomal subunit (*SSU*) is coloured in *teal*, the large ribosomal subunit (*LSU*) in *grey,* and the structural tRNA^Val^ in *black*. The positions of mammalian mt-rRNA modifications are highlighted in *various colours* and the chemical structures of the corresponding modifications are indicated

The mammalian mitochondrial ribosome (55S), which is responsible for the translation of all mt-mRNAs, is composed of a small subunit (SSU; 28S) and a large subunit (LSU; 39S; Fig. 1) [17, 30, 31]. The 28S subunit consists of 30 ribosomal proteins and the 12S rRNA, whereas the 39S subunit is composed of 52 ribosomal proteins, the 16S rRNA, and an mt-tRNA (mt-tRNA^Val^ or mt-tRNA^Phe^) that forms the structural scaffold of the central protuberance, analogous to the 5S rRNA of cytoplasmic ribosomes. Minimisation of mitochondrial rRNA sequences by many small deletions has left a core structure similar to that of bacteria and the addition of numerous mitochondria-specific ribosomal proteins not only functionally expands mitochondrial ribosomes beyond their bacterial counterparts, but also enables several key RNA–protein interactions to be replaced by protein–protein contacts. Nevertheless, the mt-rRNAs form the essential scaffold of the ribosome, including key features such as the peptidyl transferase centre (PTC) and decoding site (DCS), and correct expression, folding, and modification of the mt-rRNAs are critical for ribosome assembly and function.

Compared to their cytoplasmic and bacterial counterparts, mammalian mt-rRNAs have a low number of modified nucleotides with only ten sites identified so far (Table 1) [32, 33], which markedly contrasts to the >200 and >30 modified nucleotides present in eukaryotic cytoplasmic and prokaryotic ribosomes, respectively [25, 34]. Mapping of the positions of mt-rRNA modifications on the recent cryo-electron microscopy structures of the mammalian mitochondrial ribosome [30] has revealed that, similar to modifications in bacterial and eukaryotic cytoplasmic ribosomes, they cluster at functionally important sites within the ribosomes, such as the PTC in the LSU and the DCS of the SSU (Fig. 1).

**Table 1:** Inventory of mammalian mitochondrial rRNA modifications

rRNA	Pos.	Mod.	Enzyme	Disease associations	References
12S	429	m^5^U	*?*		[148, 149]
12S	839	m^4^C	*?*		[148, 149]
12S	841	m^5^C	NSUN4		[44]
12S	936	${\text{m}}^{6}{}_{2} {\text{A}}$	TFB1M	Type 2 diabetes, mitochondrial-associated deafness	[35, 36, 41, 43]
12S	937	${\text{m}}^{6}{}_{2} {\text{A}}$	TFB1M	Type 2 diabetes, mitochondrial-associated deafness	[35, 36, 41, 43]
16S	947	m^1^A	TRMT61B		[33]
16S	1145	Gm	MRM1		[62]
16S	1369	Um	MRM2		[61, 62]
16S	1370	Gm	MRM3		[61, 62]
16S	1397	Ψ	RPUSD4		[57–59]

The rRNA, position (Pos.), modification (Mod.), and modification enzyme are given along with reported disease associations and references, where applicable

### Small ribosomal subunit RNA modifications

The first mitochondrial rRNA modifications studied in detail were two highly conserved adenosine dimethylations close to the 3′-end of the 12S rRNA (${\text{m}}^{6}{}_{2} {\text{A936}}$ and ${\text{m}}^{6}{}_{2} {\text{A937}}$). Based on homology to the *E*. *coli* 16S rRNA methyltransferase KsgA that installs the corresponding modifications in bacterial ribosomes, TFB1M and TFB2M were identified as mammalian *S*-adenosylmethionine (SAM)-dependent methyltransferases that are capable of introducing these modifications in the mitochondrial 12S rRNA (Table 1) [35, 36]. Notably, these proteins were initially characterised as mitochondrial transcription factors [37]; however, in vivo analyses revealed that TFB1M is primarily responsible for modification of 12S rRNA, while TFB2M mainly functions as a transcription factor (reviewed in [38]). Interestingly, ribosome-binding factor A (RBFA) was recently found to bind directly to the region of the 12S rRNA that contains these dimethylations and to be required for their efficient installation [39]. Lack of ${\text{m}}^{6}{}_{2} {\text{A}}$ in the SSU rRNA of bacteria and yeast does not significantly affect SSU biogenesis, but rather, these modifications have been implicated in maintaining translation efficiency by the ribosome or conferring increased sensitivity to antibiotics [40]. Interestingly, in mammalian mitochondria, loss of TFB1M leads to decreased stability of the small ribosomal subunit and consequently prevents mitochondrial translation. It is possible that lack of stable 28S subunits reflects a role for this modification or TFBM1 in the assembly of the SSU. However, since TFBM1 can also associate with mature 28S complexes, it is also possible that, in addition to its modification function, TFBM1 directly contributes to small subunit stability, thereby ensuring that only translation competent 28S subunits containing the ${\text{m}}^{6}{}_{2} {\text{A936}}$ and ${\text{m}}^{6}{}_{2} {\text{A937}}$ modification are present in assembled 55S monosomes. The importance of these modifications in vivo is highlighted by the findings that a conditional knockout of *TFB1M* is embryonic lethal in mouse and that a tissue-specific knockout leads to loss of ${\text{m}}^{6}{}_{2} {\text{A936}}$ and ${\text{m}}^{6}{}_{2} {\text{A937}}$, lack of 28S, and impaired mitochondrial translation [41]. Furthermore, genetic analyses revealed that TFB1M is a type 2 diabetes risk gene and consistent with this, a TFB1M^+/−^ mouse model showed impaired mitochondrial translation in pancreatic islet cells and reduced insulin production in response to glucose, implying that lack of 12S modification and the consequent mitochondrial dysfunction contribute to the pathogenesis of type 2 diabetes [42, 43].

More recently, a 5-methylcytosine (m^5^C) at position 841 of the 12S rRNA has been shown to be installed by the Nol1/nop2/SUN family protein, NSUN4 [44]. While other members of this family of m^5^C methyltransferases have been shown to target tRNAs [45–49], NSUN1 and NSUN5 are implicated in m^5^C modification of rRNAs destined for cytoplasmic ribosomes [50, 51]. NSUN4 is essential for embryonic development in mice and tissue-specific conditional knockout of NSUN4 showed that in heart, lack of NSUN4 leads to progressive cardiomyopathy. Reduced levels of the OXPHOS complexes containing mt-DNA-encoded proteins but not complex II that is assembled from nuclear-encoded proteins in these mice demonstrated that NSUN4 is essential for mitochondrial translation. While it is likely that this reflects the importance of 12S-m^5^C841 modification for ribosome function, interestingly, NSUN4 has a dual function in mitochondrial ribosome biogenesis as, together with MTERF4, it is also important for LSU assembly [44, 52, 53]. Notably, its catalytic function on 12S rRNA is independent of MTERF4 and lack of NSUN4 (or m^5^C841) does not affect the installation the 12S-${\text{m}}^{6}{}_{2} {\text{A936}}$ and ${\text{m}}^{6}{}_{2} {\text{A937}}$ modifications, implying that it is not essential for assembly of the SSU. Several other methyltransferases involved in rRNA modification have also been demonstrated to have dual functions [25, 40, 54, 55]. However, these enzymes are typically required for different aspects of the biogenesis of a single ribosomal subunit, but in the case of NSUN4, it is possible that its functions in both SSU modification and LSU assembly may represent a mechanism for co-ordinating maturation of both ribosomal subunits [44]. Based on homology to hamster, the human 12S rRNA is also predicted to contain a 5-methyluridine (m^5^U) at position 429 and a 4-methylcytosine (m^4^C) at position 839, but the presence of these modifications remains to be confirmed [32].

### Large ribosomal subunit RNA modifications

In eukaryotic cytoplasmic ribosomes, the most common modifications are 2′-*O*-methylations of the ribose and the isomerisation of uridine to pseudouridine [56]. Recently, a systematic analysis of mitochondrial pseudouridine synthetases and a pseudouridine mapping approach on mitochondrial RNAs confirmed pseudouridylation of U1397 of the 16S rRNA [57], and RPUSD4 was identified as the enzyme responsible for this modification [58, 59]. Although this modification is present in both yeast and mammalian mitochondrial ribosomes, in *S*. *cerevisiae,* the modification is not essential for cell viability [60] and its precise function is not yet known.

In the mammalian mitochondrial 16S rRNA, there are also three 2′-*O*-methylations, Gm1145, Um1369, and Gm1370, which are installed by MRM1, MRM2, and MRM3, respectively (Table 1) [61–63]. These modifications lie within the A (aminoacyl)-site (Um1369 and Gm1370) and the P (peptidyl)-site (Gm1145) of the PTC (Fig. 1). 2′-*O*-methylation of the A-loop is an evolutionarily conserved feature of ribosomes that is important for mediating interactions with aminoacylated tRNAs. It has been suggested that the extent of modification of G1370 is influenced by the adjacent Um1369 modification, implying that the catalytic action of MRM2 may precede that of MRM3 [61]. This is analogous to the installation of Um2921 and Gm2922 during the biogenesis of the yeast cytoplasmic ribosomes as 2′-*O*-methylation of 25S-Um2921 (equivalent to Um1369) occurs co-transcriptionally, whereas methylation of 25S-G2922 by Spb1 is a late step in pre-LSU assembly [24, 64]. This temporal model is supported by the fact that G1370 is accessible on the surface of the mature mitochondrial ribosome. Interestingly, however, immunoprecipitation data and sucrose density gradient centrifugation analyses suggest that while both MRM2 and MRM3 associate with LSU complexes, MRM2 also interacts with mature 55S monosomes. The relevance of this finding is not clear yet, but it is tempting to speculate that, similar to NSUN4, MRM2 has dual functions in rRNA modification and mediating ribosome assembly, further supporting the model that coupling of these events serves as a checkpoint for fidelity of ribosome assembly. Depletion of MRM2 and MRM3 significantly inhibits mitochondrial translation and results in a corresponding reduction in cellular oxygen consumption rate. The precise influence of these A-loop modifications on translation is not known yet; however, lack of the modification equivalent to Um1369 in *E*. *coli* ribosomes (23S-Um2552) leads to decreased programmed +1 and −1 frameshifting and reduced read-through of UAA and UGA [65]. Interestingly, −1 frameshifting is necessary for translation termination of the COX1 and ND6 mt-mRNAs [66], suggesting that MRM2 and Um1369 may play a similar role in mitochondrial translation.

The inventory of 16S rRNA modifications was recently extended to also include a 1-methyladenosine (m^1^A) at position 947 (Table 1) [33]. Genome-wide association studies suggested a functional link between modification of this position and single-nucleotide polymorphisms in the tRNA methyltransferase TRMT61B. The action of this enzyme, which is also responsible for modification of position 58 of several mt-tRNAs (see below), in m^1^A methylation of 16S-A947, was confirmed by primer extension and RNA-seq analyses of RNA derived from cells depleted of TRMT61B [33]. In vitro methylation assays demonstrated that isolated 16S rRNA can be efficiently methylated implying that this modification is installed during the early stages of 39S biogenesis and close inspection of the sequence and secondary structural context of 16S-m^1^A947 and position 58 of the m^1^A modified tRNAs also targeted by TRMT61B revealed a weak consensus motif. The precise function of this modification in mitochondrial translation remains to be elucidated, but studies using a bacterial model system suggest that it could be important for optimal mitoribosome activity [33]. Interestingly, analysis of the presence of this modification through evolution revealed that it occurs on approximately 90% of mammalian 16S rRNA sequences, whereas the remaining 10% of mammalian mitochondrial ribosomes, and eukaryotic cytoplasmic ribosomes, carry an unmodified uridine at this position. In contrast, an unmodified guanine is present at the equivalent position in most bacterial ribosomes. m^1^A947 lies within helix 71 of the 39S subunit, which is located at the intersubunit interface in close proximity to the intersubunit bridge B3. Based on the tertiary structure of the mitoribosome, it was proposed that the positively charged m^1^A may facilitate formation of stabilising electrostatic contacts between m^1^A947 and the rRNA backbone of helix 64. It is probable, therefore, that the importance of the m^1^A947 modification lies in its contribution to maintaining the integrity of assembled 55S monosomes during translation. While unmodified adenine is unable to form such interactions, the uridine or guanine nucleotides present in cytoplasmic and prokaryotic ribosomes, respectively, likely represent alternative strategies for stabilisation of this region of the ribosome. This raises the question of why the majority of mammalian mitochondrial ribosomes rely on installation of an RNA modification by a nuclear-encoded enzyme when other unmodified nucleotides are sufficient to fulfil its function. It is possible that employment of a single enzyme for both rRNA modification and tRNA modification represents a mechanism by which different components of the translation machinery can be co-regulated. Alternatively, it has been suggested that there is a selective pressure on the presence of adenosine at this position in the mitochondrial genome as it may also contain important regulatory elements [33].

Together, the post-transcriptional modification of mt-rRNAs is important for ensuring the stability and functionality of mitoribosomes. The requirement for several mt-rRNA modifying enzymes for the stability of mature 55S ribosomes suggests the existence of quality control mechanisms, which guarantee that only mitoribosomes containing correctly modified rRNAs can engage in translation. Furthermore, the multi-functionality of several of the identified mt-rRNA modification enzymes implies that the installation of modifications is closely coupled with other aspects of mitoribosome biogenesis and mitochondrial function.

## mt-tRNA modifications

The 22 mammalian mt-tRNAs are essential adaptors for the decoding of mt-mRNAs by the mitoribosome. Endonucleolytic processing of 5′-ends of mt-tRNAs is mediated by the mitochondrial RNase P, which, in contrast to other RNase P complexes that contain a catalytic RNA and up to 10 proteins, is assembled from only three proteins MRPP1, MRPP2, and MRPP3 [67, 68]. Maturation of the 3′-ends of mt-tRNAs is initiated by the mitochondrial RNase Z homologue, ELAC2 [69, 70], followed by addition of the universally conserved CCA sequence to the 3′-termini of all tRNAs by the tRNA nucleotidyltransferase TRNT1 [71]. In addition, after aminoacylation, similar to the bacterial initiator tRNA_i_^Met^, the portion of the single mt-tRNA^Met^ destined to act in translation initiation rather than elongation undergoes formylation by the mitochondrial methionyl-tRNA formyltransferase (MTFMT) to produce mt-tRNA^fMet^ [72]. This enables specific recognition by the mitochondrial translation initiation factor MTIF2 and recruitment to the mitoribosomal P-site to initiate translation [73]. Although mt-tRNAs contain fewer modifications than their cytoplasmic counterparts [16, 74], the installation of a diverse range of RNA modifications is essential for mt-tRNA stability and function, and so far, 15 types of RNA modifications have been detected in 118 positions in different mammalian mt-tRNAs [74]. Mitochondrial tRNA modifications can be broadly classified into two groups: anticodon loop modifications that expand the decoding capacity of mt-tRNAs and regulate the fidelity of translation, and core modifications, which primarily contribute to the structural stability of mt-tRNAs, but in some cases, may influence recognition by aminoacyl-tRNA synthetases (see for example [75]).

### Modifications in the mt-tRNA body

**Fig. 2:**
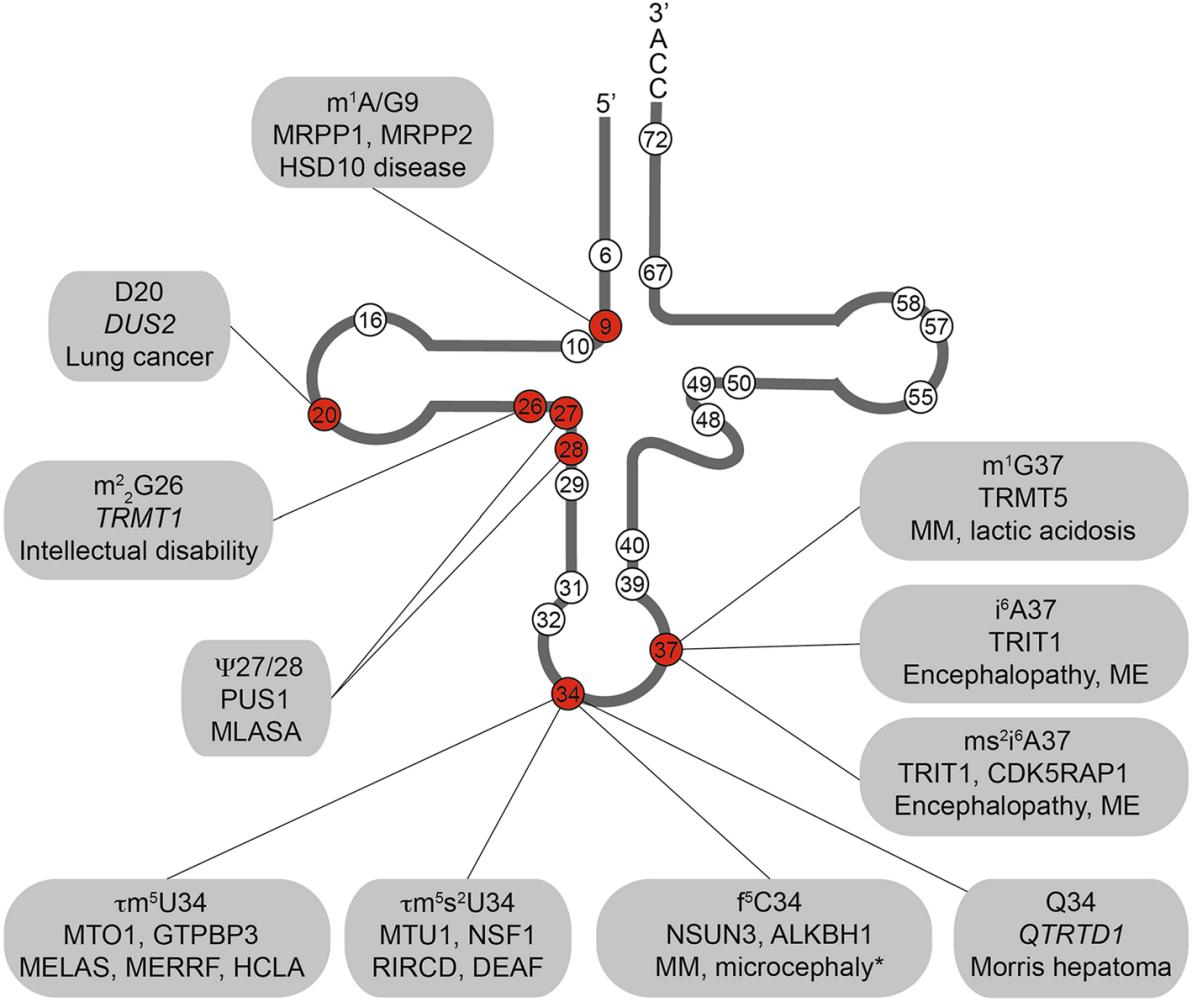
Defects in multiple mt-tRNA modification enzymes are associated with human diseases. Schematic view of the cloverleaf secondary structure of a typical tRNA on which the positions of nucleotides that are known to carry modifications in mitochondrial tRNAs are indicated with *circles*. Modifications that are associated with human diseases are indicated in *red* and the modifications present at these positions, the enzymes responsible for installing these modifications (putative modification enzymes based on homology are shown in *italics*) and the associated diseases are given. *DEAF* maternally inherited deafness; *HCLA* hypertrophic cardiomyopathy and lactic acidosis; *ME* myoclonic epilepsy; *MELAS* mitochondrial encephalomyopathy, lactic acidosis and stroke-like episodes; *MERRF* myoclonic epilepsy with ragged red fibres; *MLASA* myopathy, lactic acidosis and sideroblastic anemia; *MM* mitochondrial myopathy; *RIRCD* reversible infantile respiratory chain deficiency. *Asterisks* developmental disability, microcephaly, failure to thrive, recurrent increased lactate levels in plasma, muscular weakness, external ophthalmoplegia, and convergence nystagmus

Chemical modifications that occur in the body of mt-tRNAs (excluding positions 34 and 37) are small modifications, such as base methylations, or the conversion of uridine to either pseudouridine (Ψ) or dihydrouridine (D). Interestingly, a subcomplex of the mitochondrial RNase P consisting of MRPP1 (also known as TRMT10C) and MRPP2 (also known as HSD10 or SDR5C1) also has an endonucleolytic cleavage-independent function in the N1-methylation of purines at position 9 of many mt-tRNAs (Table 2; Fig. 2) [76]. MRPP1 is the SAM-dependent methyltransferase responsible for substrate recognition and introduction of these modifications. In contrast, while MRPP2 that can act as a dehydrogenase and contains a Rossmann-fold NAD(H) dinucleotide-binding domain is essential for m^1^A/G9 modifications, its catalytic activity is dispensable for this function and it does not significantly contribute to tRNA binding by MRPP1. Although the precise role of MRPP2 in enabling modification, therefore, remains unclear, it is likely that it influences the stability or conformation of MRPP1 to promote methylation. The presence of N1-methylation at position 9 in 19 of the 22 mt-tRNAs implies an important physiological role for this modification, and indeed, it has been shown that unmodified A9 of mt-tRNA^Lys^ basepairs with U64 leading to mis-folding of the tRNA [77–79]. The dual function of MRPP1 and MRPP2 in endonucleolytic processing of the 5′-ends of tRNA sequences and N1-methylation of A/G9 may indicate coupling of these processes, ensuring high modification efficiency of this important position. Mutations in MRPP2 have been shown to cause a disease characterised by progressive neurodegeneration and cardiomyopathy, termed HSD10 disease (Fig. 2). These pathogenic mutations not only impede dehydrogenase activity but also inhibit the interaction of MRPP2 with MRPP1, leading to decreased m^1^A/G9 modification [80]. However, given the multifunctional nature of MRPP1 and MRPP2, it is not yet clear whether the lack of tRNA modification directly contributes to HSD10 pathogenesis [81].

**Table 2:** Inventory of mammalian mitochondrial tRNA modifications

Pos.	Mod.	mt-tRNA species	Enzyme(s)	Disease associations	Refs.
6	m^2^G	Asp	*THUMPD2* or THUMPD*3*		[91]
9	m^1^A	Ala, Arg, Asp, Asn, Glu, Gly, His, Leu^CUN^, Lys, Phe, Pro, Thr, Trp, Val,	**MRPP1, MRPP2**	HSD10 disease	[76, 80]
	m^1^G	Cys, Gln, Ile, Leu^UUR^, Tyr	**MRPP1, MRPP2**	HSD10 disease	[76, 80]
10	m^2^G	Ala, Asn, Phe, Gly, His, Leu^UUR^, Leu^CUN^, Lys, Pro, Trp, Tyr, Val	*TRMT11*		[92]
16	m^1^A	Arg	*?*		[84]
20	D	Leu^UUR^, Leu^CUN^, Ser^UCN^	*DUS2*	Lung cancer	[93, 94]
26	m^2^G	Ala, Glu, Leu^UUR^	*?*		
	${\text{m}}^{2}{}_{2} {\text{G}}$	Ile	*TRMT1*	Intellectual disability	[86–88]
27	ψ	Asn, Asp, Cys, His, Ile, Leu^UUR^, Leu^CUN^, Met, Pro, Val	**PUS1**	MLASA	[98, 99]
27a	ψ	Ser^UCN^	**PUS1**	MLASA	[98]
28	ψ	Ala, Asn, Cys, Glu, Leu^CUN^, Lys, Ser^UCN^, Tyr	**PUS1**	MLASA	[98, 99]
29	ψ	Ser^UCN^	*PUS1*		[98]
31	ψ	Asp, Leu^CUN^	*RPUSD1*		[100]
32	ψ	Cys, Val	*RPUSD2*		[101]
	m^3^C	Ser^UCN^, Thr	*?*		
34	τm^5^U	Gln, Glu, Leu^UUR^, Lys, Trp	**GTPBP3, MTO1**	MELAS, MERRF, HCLA	[118, 119, 124, 125]
	τm^5^s^2^U	Gln, Glu, Lys	**GTPBP3, MTO1, MTU1**	RIRCD, DEAF	[120, 121, 127–131]
	f^5^C	Met	**NSUN3, ABH1**	MM, developmental disability, microcephaly, failure to thrive, external ophthalmoplegia, convergence nystagmus	[45, 110, 111]
	Q	Asn, Asp, His, Tyr	*QTRTD1*	Morris hepatoma	[107]
37	t^6^A	Asn, Ile, Lys, Ser^AGY^, Thr	*YRDC*, *OSGEPL1*		[135–137]
	i^6^A	Cys, Phe, Ser^UCN^, Trp, Tyr	**TRIT1**	Encephalopathy and myoclonic epilepsy, lung cancer	[140, 143]
	ms^2^i^6^A	Phe, Ser^UCN^, Trp, Tyr	**TRIT1, CDK5RAP1**	Encephalopathy and myoclonic epilepsy, lung cancer	[143, 144, 146]
	m^1^G	Gln, Leu^CUN^, Pro	**TRMT5**	Mitochondrial myopathy, lactic acidosis	[132, 134]
39	ψ	Ala, Arg, Cys, Gln, Gly, His, Leu^UUR^, Phe, Tyr	*PUS3*		[102]
40	ψ	Gln, Glu	*PUS3*		[102]
48	m^5^C	Asn, Leu^UUR^, Trp	*?*		
49	m^5^C	Glu, Ser^AGY^	*?*		
50	ψ	Met	?		
55	ψ	Gln, Glu, Ser^UCN^, Tyr	*TRUB2*		[58, 103]
57	ψ	Ala	?		
58	m^1^A	Cys, Glu, Ile, Lys, Leu^UUR^, Ser^UCN^	**TRMT61B**		[82]
67	ψ	Thr	*PUS1*		[98]
72	m^5^C	Thr	*?*		

The positions (Pos.) of modifications (Mod.) detected in bovine mt-tRNA species, according to [74] or the individual references given, are shown. Enzymes demonstrated to be involved in installing these modifications in mammals are shown in bold and predicted enzymes (based on homology to the enzymes responsible for these modifications in other species) are given in italics, along with disease associations arising from mutations in the known or predicted modification enzymes and references (Refs.) where applicable

*DEAF* maternally inherited deafness, *HCLA* hypertrophic cardiomyopathy and lactic acidosis, *MELAS* mitochondrial encephalomyopathy, lactic acidosis and stroke-like episodes, *MERRF* myoclonic epilepsy with ragged red fibres, *MLASA* mitochondrial myopathy, lactic acidosis and sideroblastic anemia, *MM* mitochondrial myopathy, *RIRCD* reversible infantile respiratory chain deficiency

m^1^A modifications have also been detected at position 58 of six bovine mt-tRNAs (Cys, Glu, Ile, Lys, Leu^UUR^, and Ser^UCN^) and TRMT61B was confirmed to be the enzyme responsible for introducing these modifications in mt-tRNA^Leu^, mt-tRNA^Lys^, and mt-tRNA^Ser^ [82]. m^1^A modifications at position 58 of tRNAs introduce additional positive charge to the T-loop, and in several tRNAs, this enhances the stability of their tertiary structure. More specifically, the cytoplasmic tRNA_i_^Met^ lacking the m^1^A58 modification was shown to be actively targeted for degradation by the exosome in yeast [83]. Interestingly, TRMT61B has also recently been shown to be responsible for N1-methylation of A947 of the 16S rRNA (see above), raising the possibility that tRNA stability is co-ordinated with ribosome functionality in mitochondria. Furthermore, primer extension analyses of the extent of m^1^A58 modification of mt-tRNA^Lys^ and mt-tRNA^Ser^ suggest that these positions may be substoichiometrically modified, implying that dynamic regulation of m^1^A58 modifications in different conditions may be a mechanism by which mitochondrial translation could be regulated [82]. This hypothesis is supported by the recent finding that the extent of N1-methylation of A58 of mt-tRNA^Lys^ is increased in cells lacking the dioxygenase ALKBH1, an enzyme which has been reported to act as an m^1^A demethylase for selected cytoplasmic tRNAs [84, 85]. Interestingly, loss of ALKBH1 was also found to increase N1-methylation of position 16 of the mt-tRNA^Arg^, a position not previously reported to carry an m^1^A modification [84].

Further base methylations detected in mt-tRNAs outside the anticodon or position 37 are *N* ^2^-methylguanosine (m^2^G) at positions 6, 10, and 26, *N* ^2^,*N* ^2^-dimethylguanosine (${\text{m}}^{2}{}_{2} {\text{G}}$) at position 26, 3-methylcytosine (m^3^C) at position 32, and 5-methylcytosine (m^5^C) at positions 48, 49, and 72 of specific mt-tRNAs (Table 2). While the enzymes responsible for installing these modifications in human mt-tRNAs have not been confirmed, some enzymes are predicted based on their homology to methyltransferases known to install corresponding tRNA modifications in other species. The in vitro methylation activity of TRMT1, its mitochondrial localisation, and its similarity to yeast Trm1 strongly implicates this enzyme in ${\text{m}}^{2}{}_{2} {\text{G}}$ synthesis at position 26 of mt-tRNA^Ile^ [86–88], and it is also anticipated to be responsible for m^2^G methylations at the corresponding position of mt-tRNA^Ala^, mt-tRNAs^Glu^, and mt-tRNA^Leu^. Importantly, mutations that cause a frameshift in TRMT1 and consequent lack of the protein have been identified in several patients with intellectual disability (Fig. 2) [89, 90]. Similarly, based on their homology to *Methanocaldococcus jannaschii* Trm14, THUMPD2 or its paralogue THUMPD3 are predicted to install m^2^G6 modifications [91], and TRMT11 is a strong candidate for m^2^G modification of position 10 of human mt-tRNAs as its yeast homologue has been shown to perform this function [92]. While studies on cytoplasmic tRNA modifications in yeast and humans provide putative candidates for the cytosine methyltransferases responsible for the m^3^C32 (METTL2B), m^5^C48 and m^5^C49 (NSUN2), and m^5^C72 (NSUN6) modifications found in some mitochondrial tRNAs, the activity of these enzymes on mitochondrial tRNAs has not been demonstrated so far and it is possible that other uncharacterised mitochondrial methyltransferases are instead responsible for installing these modifications.

In addition to the base methylations described above, tRNAs also undergo post-transcriptional modification to generate two derivatives of uridine: pseudouridine (ψ) and dihydrouridine (D). Although D is a universally conserved modified base found in tRNAs from all three domains of life and is highly abundant in eukaryotic cytoplasmic tRNAs and yeast mitochondrial tRNAs, only U20 of mt-tRNA^Leu^ and mt-tRNA^Ser^ are reported to be converted to D in human cells [74]. Although the human genome encodes four flavin mononucleotide (FMN)-dependent dihydrouridine synthetases (DUS1-4), DUS2 is likely to be responsible for the reduction of uridine to dihydrouridine in human mt-tRNAs. This conclusion is supported by the high specificity of Dus proteins for individual tRNA positions in yeast and the confirmed role of yeast Dus2 in D20 formation [93], as well as the detection of human DUS2 in mitochondria [94]. The precise role of D20 modifications in mt-tRNAs is not known, however, the increased conformational flexibility of D compared to uridine [95] suggests that this modification may contribute to folding and stability of mt-tRNA^Leu^ and mt-tRNA^Ser^. Notably, DUS2 is upregulated in many lung cancers (Fig. 2) [96], and while a corresponding increase in dihydrouridine in the cytoplasmic tRNA^Phe^ has been observed [97], it is possible that changes in DUS2 levels similarly affect mt-tRNA modification and that this may also contribute to carcinogenesis.

In contrast to the relatively rare D modifications, pseudouridines are found at positions 27, 27a, 28, 29, 31, 32, 39, 40, 50, 55, 57, and 67 of several mt-tRNAs (Table 2). The prevalence of pseudouridine in mt-tRNAs is likely to reflect the strong stabilising effect of this modification on RNA secondary structure, as the isomerisation of uridine to pseudouridine confers greater hydrogen bonding potential and enhances the rigidity of the sugar-phosphate backbone. The pseudouridine synthase PUS1 mediates formation of Ψ27 and Ψ28, and is thought to also be responsible for Ψ29 and Ψ67 in individual mt-tRNAs [98]. This multifunctional enzyme is also responsible for installing modifications at the corresponding positions of numerous cytoplasmic tRNAs, and in yeast, Pus1 has been linked to various pseudouridylations detected in cytoplasmic mRNAs by genome-wide modification mapping [99]. Importantly, genetic analyses have revealed that a missense mutation in the *PUS1* gene underlies mitochondrial myopathy and sideroblastic anemia (MLASA), an autosomal recessive, oxidative phosphorylation disorder, and lack of Ψ27 and Ψ28 modifications have been confirmed in MLASA patients (Fig. 2). The human genome encodes several further pseudouridine synthetases that localise to mitochondria and based on homology to characterised yeast tRNA modification enzymes, RPUSD1 and RPUSD2 are likely candidates for catalysing Ψ31 and Ψ32 synthesis, respectively [100, 101]. Similarly, it is anticipated that PUS3 is responsible for pseudouridylation of positions 39 and 40 in several tRNAs, and Ψ55 modifications are probably installed by TRUB2 (Table 2) [102, 103]. Interestingly, depletion of either TRUB2 or another mitochondrial pseudouridine synthetase RPUSD3 has been shown to decrease Ψ6294 in the COXI mRNA and Ψ9904-6 in the COXIII mRNA [58], implying that several of these enzymes may in fact have a broad target spectrum. Furthermore, in contrast to yeast, in humans, additional pseudouridines are present at position 57 of mt-tRNA^Ala^ and position 50 of mt-tRNA^Met^, and it is likely that these modifications are also introduced by the above-mentioned enzymes, but these activities currently remain to be assigned [74].

### Anticodon and position 37 modifications

**Fig. 3:**
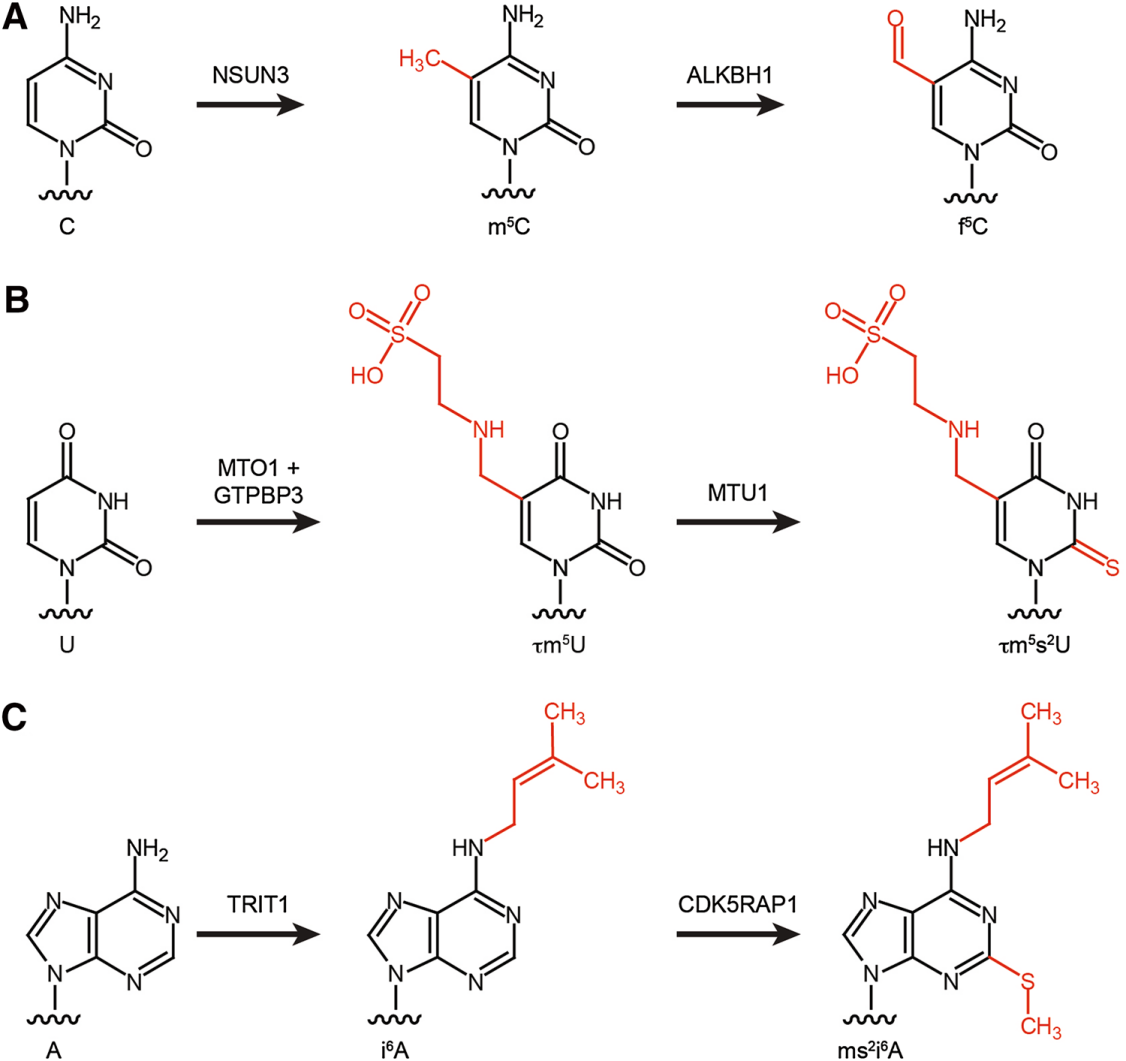
Modification pathways for selected mt-tRNA anticodon loop modifications. **a** NSUN3 methylates C5 of cytosine (C) at position 34 of mt-tRNA^Met^ to produce m^5^C, which can then be oxidised to 5-formylcytosine (f^5^C) by ALKBH1. **b** Uridine at position 34 of selected mt-tRNAs can be converted to τm^5^U by MTO1 and GTPBP3. τm^5^U can then undergo O/S exchange by MTU1 to produce τm^5^s^2^U. **c** TRIT1 isopentenylates N6 of adenosine at position 37 of several mt-tRNAs to produce *N* ^6^-isopentenyladenosine (i^6^A). CDK5RAP1 can then perform methylthiolation to generate 2-methylthio-*N* ^6^-isopentenyladenosine (ms^2^i^6^A)

Decoding of the 60 codons used in the non-universal genetic code of mammalian mitochondria by the minimal set of 22 mt-tRNAs relies on non-canonical basepairing between the first position of the tRNA anticodon (the wobble position) and the third base of the codon triplet. Post-transcriptional mt-tRNA modifications within the anticodon loop are essential to achieve this flexibility in decoding. Four types of modified nucleotides are found at the wobble position of mt-tRNAs: C34 of mt-tRNA^Met^ can be modified to 5-formylcytosine (f^5^C), queuosine (Q) is present at the wobble position of mt-tRNAs Asn, Asp, His, and Tyr, and U34 of mt-tRNAs Gln, Glu, Leu, Lys, and Trp can be modified to carry the taurine-containing modifications τm^5^U or τm^5^s^2^U (Table 2; Fig. 3).

Queuosine (Q) is a universally conserved anticodon modification that differs from most other RNA modifications in that it is not generated from one of the four basic nucleotides within the context of a cellular RNA, but rather involves the formal substitution of a guanosine for the heavily modified 7-deaza-guanosine derivative, queuosine [104]. In bacteria, queuosine can be generated in a multistep biosynthetic pathway [105], whereas in humans, queuosine is obtained either from the diet or from the intestinal microflora [106]. Insertion of Q into tRNAs is achieved by tRNA-guanine transglycosylases (TGTases), and in humans, the mitochondrially localised QTRTD1 enzyme is likely to be responsible for this modification [107]. While it has been suggested that the presence of Q at the wobble position of four tRNAs (Table 2) may contribute to anticodon–codon interactions and regulate codon selection [108, 109], the precise role of this modification remains to be elucidated.

**Fig. 4:**
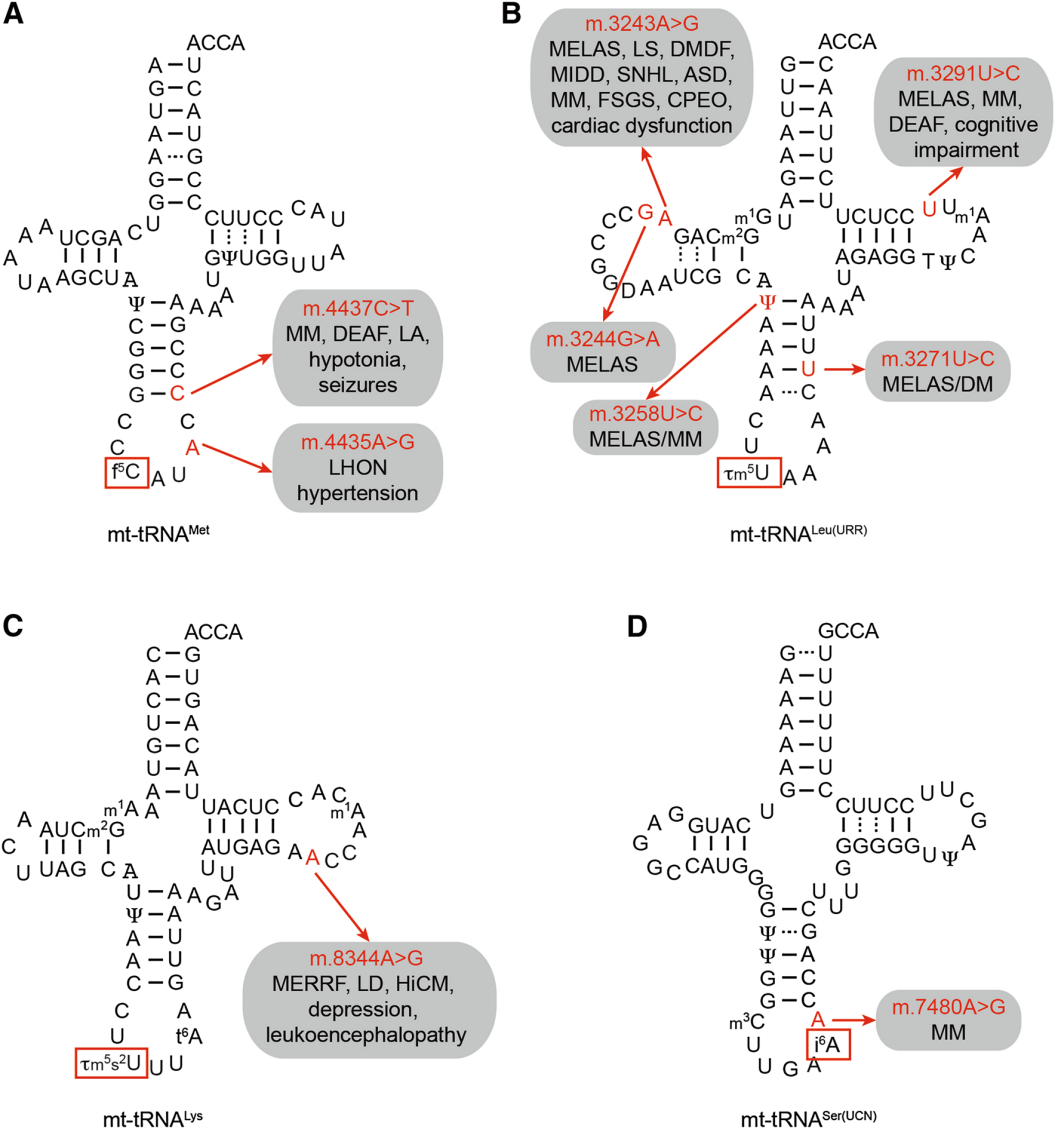
Pathogenic mutations resulting in sequence changes in mt-tRNAs can lead to decreased levels of anticodon loop modifications. Schematic view of the secondary structures of four mt-tRNAs, mt-tRNA^Met^ (**a**), mt-tRNA^Leu(URR)^ (**b**), mt-tRNA^Lys^ (**c**), and mt-tRNA^Ser(UCN)^ (**d**), with the positions of pathogenic mutations that lead to decreased levels of anticodon loop modifications (*boxed*), labelled, and highlighted in *red*. The diseases associated with each mutation are indicated. *ASD* autistic spectrum disorders; *CPEO* chronic progressive external ophthalmoplegia; *DM* diabetes mellitus; *DMDF* diabetes mellitus and deafness; *FSGS* focal segmental glomerulosclerosis; *HiCM* histiocytoid cardiomyopathy; *LA* lactic acidosis; *LHON* Leber hereditary optic neuropathy; *LS* Leigh syndrome; *MELAS* mitochondrial encephalomyopathy, lactic acidosis and stroke-like episodes; *MERRF* myoclonic epilepsy with ragged red fibres; *MIDD* maternally inherited diabetes and deafness; *MLASA* myopathy, lactic acidosis and sideroblastic anemia; *MM* mitochondrial myopathy; *DEAF* maternally inherited deafness; *SNHL* sensorineural hearing loss

In contrast to Q, f^5^C is a modification that is only present in a single metazoan mitochondrial tRNA (mt-tRNA^Met^), and recently, the modification pathway and corresponding enzymes were identified. In general, enzymatic formylation can take place via two alternative mechanisms: in a single-step reaction using formyl-tetrahydrofolate as a formyl group donor or in a two-step reaction involving oxidation of a pre-installed methyl group. In the case of mt-tRNA^Met^, cytosine 34 is first methylated at position 5 of the pyrimidine ring by the methyltransferase NSUN3 [45, 110–112] and this m^5^C then undergoes oxidation by the Fe(II) and α-ketoglutarate-dependent dioxygenase ALKBH1 (also known as ABH1) to form f^5^C (Fig. 3a) [45, 112]. Cytosine 34 of mt-tRNA^Met^ is almost fully modified, and while both mass spectrometry and bisulfite sequencing confirm the predominance of f^5^C at this position, two independent studies also detected m^5^C34 in vivo, suggesting that a fraction of mt-tRNA^Met^ may not be oxidised by ALKBH1 [45, 110, 111]. Notably, while other dioxygenases, such as the TET proteins, generate f^5^C in DNA via a stable 5-hydroxymethylcytosine (hm^5^C) intermediate [113], ALKBH1 generates predominantly f^5^C. In cytoplasmic translation, two alternative tRNA^Met^ are required to read the classical AUG codon during translation initiation and elongation, whereas, due to the non-conventional genetic code of human mitochondria, the single mt-tRNA^Met^ is employed for decoding of AUG, AUA and AUU codons during initiation, and AUG and AUA codons during elongation. The f^5^C modification is proposed to enhance the structure and thermodynamic properties of the anticodon [114, 115], and to facilitate the increased decoding capacity by shifting the tautomeric equilibrium of the wobble base cytosine towards the imino-oxo tautomer enabling basepairing with adenine in the third codon position [116]. Interestingly, the decoding capacity of the mt-tRNA^Met^ is specifically regulated in the context of the ribosome. During translation initiation, the AUU initiation codon in the NADH dehydrogenase 2 (ND2) mRNA is recognised by mt-tRNA^Met^ leading to incorporation of methionine as the first amino acid, while during elongation, mt-tRNA^Ile^ is recruited to AUU codons for the incorporation of isoleucine, following the universal genetic code. Since both the mitochondrial translation initiation factor MTIF2 and the mitochondrial translation elongation factor TUFM can deliver mt-tRNA^Met^ to mitoribosomes, these findings indicate fine differences in tRNA selection and decoding of the AUU codon between P-site (initiation) and A-site (elongation), which will be interesting to explore on the structural level. The importance of this modification pathway is further supported by the finding that lack of NSUN3 or ABH1 leads to decreased mitochondrial translation in various cell lines. Furthermore, the integrity of the anticodon stem-loop of mt-tRNA^Met^ is essential for recognition by NSUN3 and mutations that disrupt the stability of the ASL (e.g., m.4435A>G and m.4437C>T) have been identified in patients with various diseases associated with mitochondrial dysfunction, implying that lack of this modification can be the molecular basis of these pathologies (Fig. 4a) [45, 110]. Similarly, a patient with developmental disabilities, microcephaly, muscle weakness, and ophthalmoplegia was found to carry heterozygous loss-of-function mutations in NSUN3, further highlighting the importance of this modification for mitochondrial function (Fig. 2) [111].

Thirteen of the twenty-two mitochondrial tRNAs have uridine encoded at position 34, and while eight remain unmodified at this site, five carry taurine-containing modifications (τm^5^U in mt-tRNA^Leu^ and mt-tRNA^Trp^ and τm^5^s^2^U in mt-tRNA^Lys^, mt-tRNA^Glu^, and mt-tRNA^Gln^; Fig. 3b). The high frequency of uridines at the wobble base of mt-tRNA is due to the high conformational flexibility of U, which enables it to basepair with all four nucleotides at the third codon position (the four-way wobble rule or “super-wobbling”) meaning that the eight mt-tRNAs carrying unmodified uridines are able to decode more than half of the codons used in the mitochondrial genetic code [117]. In contrast, mt-tRNAs responsible for decoding only two codons ending in purines (NNA/G) carry τm^5^(s^2^)U modifications, which fix the uridine in the C3′-endo form, strongly favouring basepairing with purines rather than pyrimidines. The use of taurine in these mt-tRNA wobble base modifications is specific for metazoa as in bacterial and yeast mitochondrial tRNAs, U34 is modified to 5-carboxymethylaminomethyluridine (cmnm^5^U). However, while the reason for this evolutionary difference is unclear, the mechanisms utilised to generate both cmnm^5^U and τm^5^U are thought to be similar and involve homologous enzymes. In yeast mitochondria, Mss1 and Mto1 form a heterodimer that is responsible for the synthesis of cmnm^5^U using glycine as a substrate and FAD and GTP as cofactors. The human mitochondrial proteins GTPBP3 and MTO1 are able to complement for lack of Mss1 and Mto1 in yeast, strongly suggesting that they perform the analogous modifications in humans [118, 119], although their activity in human cells has not formally been demonstrated. In the case of mt-tRNA^Lys^, mt-tRNA^Gln^, and mt-tRNA^Glu^, the τm^5^U34 can be further modified to τm^5^s^2^U. A thiol group is derived from l-cysteine by the partly mitochondrial cysteine desulfurase NFS1 [120], and is transferred to the 2-thiouridylase MTU1 that is responsible for thiolation of the C2 position of τm^5^U to form τm^5^s^2^U (Fig. 3b) [121]. Structural studies together with analysis of mutated mt-tRNAs in an in vitro mammalian mitochondrial translation system have revealed that the τm^5^U34 modification is necessary for decoding UUG codons as it serves to stabilise U:G wobble basepairing by increasing stacking interactions [122, 123]. Interestingly, a number of point mutations in mt-tRNA^Leu(UUR)^ (m.3243A>G, m.3244G>A, m.3258T>C, m.3271T>C and m.3291T>C) detected in patients with mitochondrial encephalomyopathy, lactic acidosis, and stroke-like episode (MELAS) have been correlated with a lack of τm^5^U34 modification (Fig. 4b) [26, 124, 125]. This implies that nucleotide substitutions at these positions in the D-, and T-loops and anticodon stem, impede recognition of mt-tRNA^Leu(UUR)^ by the MTO1-GTPBP3 heterodimer, either directly or indirectly by causing mis-folding of the mt-tRNA. Consistent with the role of this modification in facilitating decoding of UUG codons, a specific decrease was also observed in the expression of the complex I component ND6, which is expressed from an mRNA rich in UUG codons. Since a reduction in complex I activity is characteristic of MELAS and a mutation in the ND6 mt-mRNA itself (m.14453A>G) is also associated with this syndrome [126], it is highly likely that a lack of complex I caused by defective τm^5^U34 modification of mt-tRNA^Leu(UUR)^ can be the pathogenic basis of this mitochondrial disorder. Similarly, an m.8344A>G mutation in mt-tRNA^Lys^ that is associated with myoclonic epilepsy with ragged red fibres (MERRF) syndrome causes a lack of τm^5^s^2^U34 modification (Fig. 4c) [127]. On a molecular level, τm^5^s^2^U34 modifications have been found to be essential for decoding of AAR (R = A or G) codons and, consistent with this, a general defect in mitochondrial translation was observed in cells expressing the m.8344A>G mutation. The importance of the τm^5^U34 and τm^5^s^2^U34 modifications is further underlined by the finding that not only mutations in the mt-tRNAs carrying these modifications, but that also mutations in the enzymes that install them can cause disease. Mutations in both MTO1 and GTPBP3 cause mitochondrial translation defects and hypertrophic cardiomyopathy, lactic acidosis, and encephalopathy [128–130], whereas defects in the 2-thiouridylase MTU1 are implicated in reversible infantile liver injury (Fig. 2) [131].

Similar to the wobble base, position 37 within the anticodon loop of tRNAs is a hotspot of RNA modifications and can carry a diverse range of complex modifications. In all human mt-tRNAs, position 37 is a purine, and in the case of mt-tRNA^Gln^, mt-tRNA^Leu^, and mt-tRNA^Pro^, the guanosine can be methylated by the methyltransferase TRMT5 [132]. This m^1^G modification plays an important role in maintaining the efficiency and accuracy of translation. More specifically, N1-methylation of G37 reduces its Watson–Crick basepairing potential, which, on the one hand, helps to maintain an open conformation of the anticodon loop by preventing interactions with other nearby mt-tRNA nucleotides, and on the other hand, also impedes erroneous interactions with mRNAs that would result in +1 frameshifting. Indeed, in bacterial tRNAs, lack of m^1^G37 modifications has been shown to lead to increased frameshifting errors [133]. The physiological importance of m^1^G37 modification was further highlighted by the finding that two non-related individuals presenting with lactic acidosis, muscle weakness, and other characteristic symptoms of mitochondrial respiratory chain complex deficiencies both carried a heterozygous mutation in *TRMT5* [134]. This mutation leads to expression of either a truncated, non-functional protein or TRMT5 in which arginine 291 was substituted for histidine, disrupting intramolecular interactions that are important for the catalytic activity of the protein. Consistent with this, the extent of methylation of G37 of mt-tRNA^Leu(CUN)^ was significantly decreased in these patients, and the observation that re-expression of wild-type TRMT5 could rescue mitochondrial respiratory function strongly suggests that lack of m^1^G37 modification is the basis of disease in these patients (Fig. 2) [134].

In contrast to the G37, adenosines at position 37 of mt-tRNAs can be modified in a range of different ways. In the mt-tRNAs Asn, Ile, Lys, Ser^AGY^, and Thr, threonylcarbamoyl adenosine (t^6^A) has been identified at position 37 [74]. While the enzymes responsible for installing this modification have not formally been identified in human cells, YRDC and OSGEPL1 are strong candidates based on their homology to the yeast Sua5 and Qri7 enzymes that introduce the corresponding modification into yeast mt-tRNAs [135–137]. Similar to m^1^G37 modifications, on a molecular level, t^6^A at position 37 has been shown to help maintain an open loop structure of the anticodon. Moreover, t^6^A37 modifications contribute to base-stacking with the first nucleotide of mRNA codons, leading to increased anticodon–codon basepairing and facilitating efficient and accurate translation [138]. While the impact of t^6^A modifications on mitochondrial translation has not been analysed so far, lack of t^6^A modifications in yeast cytoplasmic tRNAs was found to promote translation initiation at upstream non-AUG codons, increase frameshifting, and optimise the translation elongation rate by slowing elongation at codons decoded by high abundance tRNAs and accelerating translation of codons decoded by rare tRNAs [139].

The other modifications present at position 37 of mt-tRNAs involve isopentenylation of N6 of adenosine. The cytoplasmic and mitochondrial isopentenyltransferase TRIT1, which was first identified as a tumour suppressor in lung cancer [140], has been demonstrated to be responsible for introducing these modifications into a number of mt-tRNAs (see Table 2; Fig. 3c). In fission yeast, i^6^A37 modifications have been suggested to enhance the decoding stringency of cytoplasmic tRNAs leading to increased translation efficiency [141]. Notably, in cytoplasmic tRNAs, i^6^A37 modifications were found to be a co- or pre-requisite for installation of m^3^C32 modifications, suggesting that in some cases, the installation of anticodon loop modifications is co-ordinated [142], but it remains to be seen if this is also the case in human mitochondria. In humans, pathogenic mutations that cause an arginine 323 to glutamine substitution in TRIT1 or an adenosine to guanosine switch at position 38 of mt-tRNA^Ser(UCN)^ (m.7480A>G) were found to inhibit i^6^A37 modification, suggesting that lack of mt-tRNA isopentenylation can be the basis of disease (Figs. 2, 4d) [143].

Four of the five i^6^A-containing mt-tRNAs (mt-tRNA^Phe^, mt-tRNA^Ser^, mt-tRNA^Trp^, and mt-tRNA^Tyr^) can also undergo subsequent methylthiolation to carry ms^2^i^6^A37 modifications (Fig. 3c). Based on its homology to the cytoplasmic ms^2^t^6^A methylthiotransferase, CDK5RAP1 was identified as the enzyme responsible for 2-methylthiolation of these mt-tRNAs. Interestingly, CDK5RAP1 also acts on cytoplasmic tRNAs and regulates the activity of the cyclin-dependent protein kinase (CDK5), implying that its activity is distributed between a range of substrates [144]. In bacteria, the thiomethyl group of the ms^2^i^6^A modification has been shown to stabilise A:U basepairing between the anticodon and the first base of UNN codons by inter-strand stacking [145], and similarly, in humans, reporter assays have demonstrated that ms^2^ modifications are critical for the accurate decoding of wobble codons corresponding to mt-tRNA^Phe^, mt-tRNA^Tyr^, and mt-tRNA^Ser^. Analysis of CDK5RAP1 function in *CDK5RAP1* knockout mice revealed impaired mitochondrial integrity and protein synthesis as well as accelerated myopathy and cardiac dysfunction in stress conditions [146]. Furthermore, a MELAS-associated point mutation in the sequence encoding mt-tRNA^Leu(UUR)^ (m.3243A>G) decreased ms^2^ modification. Notably, quantitative analysis of ms^2^ levels in patient samples correlated with the heteroplasmy level of the mt-DNA mutations, providing strong evidence that lack of 2-methylthiolation of mt-tRNAs contributes to this disease [146, 147].

## Concluding remarks and outlook

The mitochondrial epitranscriptome is emerging as a key regulator of organellar gene expression, and due to the special features of mammalian mitochondrial gene expression, such as the use of a non-conventional genetic code, the limited number of mt-tRNAs, which often form non-canonical structures, and the minimal mt-rRNA content of the mitoribosome, RNA modifications play especially important roles in enabling efficient, accurate, and dynamic protein synthesis in mitochondria. Systematic analyses of *Bos taurus* (bovine) mt-tRNA and *Mesocricetus auratus* (hamster) mt-rRNA modifications [74, 148, 149] have provided inventories of the core RNA modifications of the mammalian mitochondrial translation machinery and genome-wide identification of the binding sites of putative modification enzymes coupled with strategies for detection of a range of different types of modifications has increased our knowledge of the enzymes responsible for introducing these modifications. Excitingly, transcriptome-wide approaches for the mapping of RNA modifications including pseudouridine, *N* ^6^-methyladenosine (m^6^A), m^1^A, and m^5^C have recently been developed, and in addition to the detection of modifications in cytoplasmic mRNAs, intriguingly, pseudouridines have also been found in several mt-mRNAs. This not only implies that modifications in nuclear-encoded mRNAs may influence the levels of mitochondrial modification enzymes but also that the concept of dynamic regulation of gene expression by alterations in mRNA modifications also extends to mitochondria. It will, therefore, be very interesting to discover which other modifications are present in mt-mRNAs and to determine how such mRNA modifications influence the mitochondrial proteome.

Our increasing knowledge of the enzymes responsible for installing mt-RNA modifications has highlighted the fact that many of these enzymes are multifunctional. Some enzymes modify different types of mitochondrial RNA substrates, such as TRMT61B, which methylates both A947 of the 16S mt-rRNA and position 58 of several mt-tRNAs. Utilisation of the same enzyme for both tRNA and rRNA modifications could suggest that biogenesis of different components of the translation machinery is co-ordinated. Alternatively, several mt-RNA modifying enzymes are involved in other aspects of mt-RNA metabolism, such as mt-DNA transcription, mt-RNA processing, and mitoribosome assembly. This dual functionality of mitochondrial proteins extends beyond mt-RNA modification enzymes and likely reflects minimisation of the mitochondrial proteome during transfer of many genes to the nuclear DNA. This means that the installation of mitochondrial RNA modifications is closely coupled with other processes, and in some cases, this appears to have the advantage that only correctly modified RNAs can be utilised in translation, thereby acting as a quality control mechanism. Alternatively, several modifications enzymes, including PUS1, TRIT1, and CDK5RAP1, have been found to target both mitochondrial and cytoplasmic RNAs, implying that crosstalk also occurs between protein synthesis machineries in these different compartments. Such co-regulation of mitochondrial and cytoplasmic gene expression also extends to include the fact that all the enzymes involved in post-transcriptional modifications of mt-RNAs are translated on cytoplasmic ribosomes and imported into mitochondria. Interestingly, it was shown recently that production of the mitochondrial and nuclear-encoded components of the OXPHOS system is not co-ordinated at the level of transcription, but rather, that mitochondrial and cytoplasmic translation are synchronously regulated to ensure equal expression of these components [150]. This raises the possibility that differential expression of nuclear-encoded modification enzymes may be an important level of regulation of mitochondrial gene expression.

Such changes in the levels of mitochondrial RNA modification enzymes may not only regulate the extent of modification at certain sites, but since several modifications at key positions in mt-tRNAs are installed in two-step pathways via stable intermediates, this may also alter the ratio between the types of modification found at one position. The most prominent examples of such modifications are f^5^C, which is installed via m^5^C, τm^5^s^2^U that is a derivative of τm^5^U, and ms^2^i^6^A that is produced by methylthiolation of i^6^A (Fig. 3). In the case of the taurine-containing and isopentenyl-containing modifications, some mt-tRNA species have only been observed to carry either τm^5^U (mt-tRNA^Leu^ and mt-tRNA^Lys^) or i^6^A (mt-tRNA^Cys^), perhaps suggesting that they represent poor substrates for the second modification enzymes (MTU1 and CDK5RAP1), while the presence of both forms of the modifications have been detected in other mt-tRNA species (see Table 2). Similarly, while mass spectrometry-based approaches have detected only f^5^C at position 34 of mt-tRNA^Met^, recent bisulfite sequencing data from two independent studies suggest that some m^5^C may also be present at this site. Given the critical roles of these modifications in expanding and regulating the decoding capacity of mt-tRNAs, as well as ensuring the fidelity and efficiency of mitochondrial translation, a dynamic equilibrium in the proportions of these tRNAs that undergo hypermodification, could influence mitochondrial protein synthesis. For example, it is tempting to speculate that alterations in the extent of oxidation, methylthiolation, or O/S exchange of the mt-tRNAs carrying these modifications may influence the expression of particular mt-mRNAs in different conditions.

Mitochondria serve as the “power-houses” of the cell, and as such, dynamic regulation of their activity needs to be closely coupled with the cell’s metabolic status and a growing body of evidence suggests that RNA modifications may play important roles in co-ordinating the rate of mitochondrial protein synthesis with the energy needs of the cell [151]. The building blocks of several RNA modifications present in mitochondrial RNAs are harvested from metabolic pathways (e.g., taurine and queosine) and many mt-RNA modifying enzymes rely on metabolites as cofactors for their reactions. For example, the dioxygenase ALKBH1 requires α-ketoglutarate that is produced in mitochondria during the Krebs cycle, the dihydrouridine synthetases DUS2 uses FMN as a cofactor, dimethylallyl pyrophosphate (DMAPP) is used by TRIT1 for isopentenylation, and all methyltransferases characterised in mitochondria so far use *S*-adenosylmethionine as a methyl group donor. It is, therefore, a compelling hypothesis that under conditions where nutrient resources are limited, lack of mt-RNA modifications may decrease mitochondrial translation rates to conserve cellular energy, and it will be exciting to discover if such a mechanism exists. More specifically, cysteine is necessary for 2-methylthiolation of several mt-tRNAs by MTU1 and, based on work in human cell lines, it has been suggested that supplementation with cysteine may rescue mitochondrial function in cases of reversible infantile respiratory chain deficiency (RIRCD), which is caused by lack of τm^5^s^2^U modifications [152].

The high specificity of most modification enzymes is likely achieved by their recognition of defined structural features of their substrates. Given the large number of mt-DNA mutations in sequences encoding mt-tRNAs and mt-rRNAs (see MITOMAP), it is anticipated that many pathogenic mutations affect the folding of mt-tRNA or mt-rRNAs, inhibiting their recognition by modification enzymes. Indeed, several examples already exist, including m.4435A>G and m.4437C>T in mt-tRNA^Met^ that strongly affect methylation by NUSN3, m.74480A>G in mt-tRNA^Ser(UCN)^ that causes a loss of i^6^A37 modification by TRIT1 and several mutations in mt-tRNA^Leu(UUR)^ and mt-tRNA^Lys^ that prevent introduction of taurine-containing modifications (Fig. 4) [45, 110, 143, 153]. Similarly, next-generation whole-exome sequencing analysis of many patients presenting with maternally inherited deafness have been found to have mutations in mt-RNR1, which encodes the 12S rRNA. While many such mt-rRNA mutations do not affect sites that are modified, two frequently occurring mutations, m.1555A>G and m.1494C>G, lie within the ribosomal A-site, adjacent to the aminoacyl-mt-tRNA binding site, and it is suggested that the conformational changes induced by the presence of alternative nucleotides as these sites may affect the efficiency and/or accuracy of mitochondrial translation. This model is supported by the finding that several nuclear-encoded mitochondrial modification enzymes are genetically linked to these 12S mt-rRNA mutations, suggesting that they are physiological effectors of the mutations. These enzymes include TFBM1 that modifies the nearby 12S-${\text{m}}^{6}{}_{2} {\text{A936}}$ and 12S-${\text{m}}^{6}{}_{2} {\text{A937}}$ residues as well as MTO1, GTPBP3, and MTU1 that introduce τm^5^(s^2^)U modifications into the anticodons of mt-tRNAs [154–156]. As well as mt-DNA mutations, the increasing number of pathogenic mutations that have been identified in nuclear encoded modification enzymes further confirms that defects in mitochondrial RNA modification often lead to disease.

Taken together, the concomitant advancements in techniques for the detection of RNA modifications, the transcriptome-wide identification of the target sites of modification enzymes, and whole-exome sequencing of patient material now pave the way for both the physiological roles of mt-RNA modifications to be elucidated and the molecular basis of mitochondrial disorders to be understood.

## Abbreviations

A-site: Aminoacyl-tRNA-binding site on the ribosome
ATP: Adenosine triphosphate
cmnm^5^U: 5-Carboxymethylaminomethyluridine
D: Dihydrouridine
DCS: Decoding centre
f^5^C: 5-Formylcytosine
hm^5^C: 5-Hydroxymethylcytosine
i^6^A: *N* ^6^-Isopentenyladenosine
LSU: Large ribosomal subunit
m^1^A: 1-Methyladenosine
m^1^G: 1-Methylguanosine
m^2^G: *N* ^2^-Methylguanosine
$${\text{m}}^{2}{}_{2} {\text{G}}$$: *N* ^2^,*N* ^2^-Dimethylguanosine
m^3^C: 3-Methylcytosine
m^4^C: *N* ^4^-Methylcytosine
m^5^C: 5-Methylcytosine
m^5^U: 5-Methyluridine
m^6^A: *N* ^6^-Methyladenosine
$${\text{m}}^{6}{}_{2} {\text{A}}$$: *N* ^6^,*N* ^6^-Dimethyladenosine
ms^2^i^6^A: 2-Methylthio-*N* ^6^-isopentenyladenosine
MELAS: Mitochondrial encephalomyopathy, lactic acidosis and stroke-like episodes
MERRF: Myoclonic epilepsy with ragged red fibres
MLASA: Myopathy, lactic acidosis, and sideroblastic anemia
mt-DNA: Mitochondrial DNA
OXPHOS: Oxidative phosphorylation
P-site: Peptidyl-tRNA-binding site on the ribosome
PTC: Peptidyl transferase centre
Ψ: Pseudouridine
Q: Queosine
RIRCD: Reversible infantile respiratory chain deficiency
rRNA: Ribosomal RNA
SAM: *S*-Adenosylmethionine
SSU: Small ribosomal subunit
t^6^A: *N* ^6^-Threonylcarbamoyladenosine
τm^5^U: 5-Taurinomethyluridine
τm^5^s^2^U: 5-Taurinomethyl-2-thiouridine
tRNA: Transfer RNA
